# Integrated learning: ways of fostering the applicability of teachers’ pedagogical and psychological knowledge

**DOI:** 10.3389/fpsyg.2015.00738

**Published:** 2015-06-02

**Authors:** Nora Harr, Andreas Eichler, Alexander Renkl

**Affiliations:** ^1^Department of Psychology, University of FreiburgFreiburg, Germany; ^2^Department of Mathematics, University of Education FreiburgFreiburg, Germany

**Keywords:** teacher education, general pedagogical/psychological knowledge, pedagogical content knowledge, instructional design, experimental design

## Abstract

In teacher education, general pedagogical and psychological knowledge (PPK) is often taught separately from the teaching subject itself, potentially leading to inert knowledge. In an experimental study with 69 mathematics student teachers, we tested the benefits of fostering the integration of pedagogical content knowledge (PCK) and general PPK with respect to knowledge application. Integration was fostered either by integrating the contents or by prompting the learners to integrate separately taught knowledge. Fostering integration, as compared to a separate presentation without integration help, led to more applicable PPK and greater simultaneous application of PPK and PCK. The advantages of fostering knowledge integration were not moderated by the student teachers’ prior knowledge or working memory capacity. A disadvantage of integrating different knowledge types referred to increased learning times.

## Introduction

In recent years, educational research has increasingly focused on teacher competencies to enhance teaching and learning in schools (Cochran-Smith and Zeichner, 2005). In particular, being able to provide productive learning opportunities proved to be important for students’ learning outcomes (Seidel and Shavelson, 2007; Hattie, 2009). Against the background of an imbalance between university education and later job demands (Finn, 2001; Grossman, 2008), questions about how to improve teacher education have been intensively discussed (e.g., Ball, 2000; Darling-Hammond, 2006; Zeichner, 2012).

Good teachers must possess subject matter knowledge [i.e., *content knowledge* and *pedagogical content knowledge* (*PCK*); cf. Kleickmann et al., 2013] and *general pedagogical and psychological knowledge* (*PPK*; Grossman and McDonald, 2008; Voss et al., 2011). Current curricula usually treat these two knowledge types separately. On the one hand, there are courses addressing pedagogical and psychological knowledge that should be applicable across different teaching subjects (e.g., Voss et al., 2011). On the other hand, there are courses addressing the content of teaching or subject didactics (e.g., mathematics or science education). Maybe, there is the implicit hope that each student teacher will integrate the different knowledge types individually, for example, by practice teaching. Such integration, however, can be assumed to be a genuine challenge for student teachers and is therefore rarely achieved (Ball, 2000).

As a consequence, beginning teachers’ PPK, content knowledge, and PCK (Shulman, 1986) might remain largely separated in different knowledge compartments without substantial cross-references. Such compartmentalization is regarded as one factor causing inert knowledge (Whitehead, 1929; Renkl et al., 1996). In other words, general PPK, needed to optimize teaching situations (Voss et al., 2011), will barely be retrievable for teaching in certain subjects because there are only few connections to it.

We present an experimental study of the effects of providing PCK and general PPK either in a separated manner or with one out of two instructional procedures that aim to foster knowledge integration: integrating the contents by interleaving them or by prompting the learners to integrate separately taught contents. The motivation for this study was to test and compare different means of integration, whereby the second (prompted integration) seemed to be especially promising for an implementation in real-world settings, because, unlike the interleaved integration, university courses would not need to be restructured. Overall, we compared student teachers’ performances in three computer-based teaching conditions.

### Integrating Teacher Knowledge

Research on teacher knowledge was greatly inspired by Shulman (1986, 1987) who identified content knowledge, PCK, and pedagogical knowledge as founding categories of teachers’ knowledge. Since the 1980s, the distinction made between these knowledge types has become a cornerstone in research on teachers (e.g., Grossman and Richert, 1988; Borko and Putnam, 1996; Bullough, 2001; Voss et al., 2011). Competent teaching is regarded as a complex interaction between knowledge from various sources (Segall, 2004; Ball et al., 2008). Recently, Voss et al. (2011) analyzed various models of school learning in order to sharpen and unify the conceptualization of general pedagogical knowledge. In order to account for the psychological and pedagogical aspects of pedagogical knowledge, they broadened Shulman’s original definition. They expanded general pedagogical knowledge that “appear[s] to transcend subject matter” (Shulman, 1987, p. 8) in order to include pedagogical and psychological aspects and specified its components (i.e., classroom management, teaching methods, classroom assessment, knowledge about learning processes and individual characteristics). To this end, they introduced the term *general pedagogical/psychological knowledge*, including the knowledge of teaching-learning factors that are applicable across different teaching subjects and extending Shulman’s narrower conception of pedagogical knowledge.

Teachers should use their knowledge of pedagogical and psychological origins in the classroom (Shulman, 1987; Borko and Putnam, 1996; Ball et al., 2008) in combination with their content knowledge and PCK. However, this multi-component and interactive perspective of teacher knowledge did not typically translate into corresponding teacher education practice. When looking at teacher education at universities, for example, in Germany and in the USA, we usually find the curricula separated into different knowledge domains (e.g., Kennedy et al., 2008; Universität Freiburg, 2014). On the one hand, we find subject matter knowledge (i.e., content knowledge and PCK) of academic disciplines corresponding to the school subjects, and on the other hand, we find PPK taught in pedagogy courses dealing with methods of teaching and educational psychology. These pedagogical and psychological methods courses frequently make no reference to the contents of teaching or subject didactics (e.g., PCK in mathematics). However, according to Voss et al. (2011), bringing this general PPK to bear is crucial to creating productive learning situations in each subject.

Teacher education consisting of different university courses is often provided by different departments without stressing the connections to subject contents; this procedure runs the risk of student teachers encoding their knowledge in different compartments, with little if any reference to each other. In that case, the acquired knowledge may remain inert. However, such inert PPK need not always hinder good teaching. When teachers act in familiar content areas for which they have sophisticated PCK (Shulman, 1986; Borko and Putnam, 1996), such compartmentalization need not be a significant barrier to good teaching. However, when they act in unfamiliar content areas for which they lack sophisticated PCK, they seem to draw on their general PPK (Hashweh, 1987). Making use of this more general PPK can be very demanding if the educational principles (i.e., general knowledge) are taught with little or even no connection to their application in specific subjects (e.g., mathematics); transformation into effective action within an application domain will be likely to fail (Renkl et al., 1996).

This lack of applicability of more general knowledge needed for teaching is not only present in the acquisition of PPK but has also received attention from related research. A framework called *technology, pedagogy, and content knowledge* (short: TPACK) has evolved over the past decade, addressing the comparable question of how to effectively integrate knowledge on technology (Mishra and Koehler, 2006; Koehler et al., 2013). As in the case of PPK, the common approach for technology in higher education is to relegate it to separate courses and to leave the challenge of integration to the individual student teacher (Koehler et al., 2014). Within the TPACK framework, various attempts to integrate knowledge have been examined, showing beneficial effects for technology application in teaching if this knowledge is acquired in an integrated way (e.g., Koehler and Mishra, 2005; Koehler et al., 2007; Niess et al., 2010).

In accordance with TPACK research, we see a promising approach to fostering applicable PPK in crosslinking general education principles (i.e., general PPK) and subject-related topics. We assume that through this procedure, student teachers should learn where and when general pedagogical and psychological principles can be applied. They would be provided with general principles and content-specific educational examples of their application in a given content area (e.g., fractions in the case of mathematics).

An integrated teaching approach may have two major benefits. First, it provides students with educational principles enriched with content-specific examples, which fosters integrated encoding. This integration, in turn, enhances the applicability of general principles (Renkl, 2014). Second, integration can foster the simultaneous retrieval of realms of knowledge previously associated with each other (Anderson and Lebiere, 1998). Thus, the retrieval of content-specific knowledge could trigger the retrieval of general PPK when handling teaching demands.

Despite the potential benefits of integration, there may also be disadvantages. Having to integrate different types of information can induce high cognitive load and may even overwhelm (e.g., Ainsworth et al., 2002; Ayres, 2013; Schwonke et al., 2013). Learners need to allocate and regulate their cognitive resources adequately during learning in order to productively use information that must be considered simultaneously. Thus, if topics are presented in a complex, integrated way, the student must switch back and forth between perspectives (e.g., general PPK versus PCK) in order to form a coherent mental representation. In some cases, the cognitive demands affiliated with integrating different types of information can exceed the learners’ working memory capacities (Sweller et al., 2011; Schwonke et al., 2013).

If learners who possess different knowledge levels are confronted with the same task, those with little prior knowledge are more likely to be overwhelmed (Sweller et al., 1998; Ayres, 2013). Novice learners are constrained to handling information units as individual entities (Kalyuga, 2008). Compared to their ‘high-cost’ processing, proficient learners pursue a different strategy. Due to schema acquisition, they can aggregate information units into *chunks*, combining the single units into one meaningful entity then treatable as one (Ericsson and Kintsch, 1995; Kalyuga, 2008). Chunks can become highly complex and provide the basis for higher cognitive skills (Sweller et al., 1998). As a consequence of this varying facility in “chunking” information, particularly learners with little prior knowledge may experience very high or excessive cognitive load when working on a complex learning environment requiring the integration of different knowledge types (i.e., PCK and general PPK).

Besides these knowledge-dependent learning prerequisites, knowledge-independent factors may be at work as well (for an overview, see Diamond, 2013). Learners can differ in their general ability to control attention in order to keep required information in an active and quickly retrievable state (Engle, 2002). Differences in individual ability have proven to affect performances of cognitive tasks (for an overview, see Baddeley, 2012). Working memory capacities can thus be regarded as an important factor when working on complex instructional environments in which knowledge types alternate and often interrupt each other. In order to benefit from integrated learning environments where knowledge is intertwined, students must possess sufficient working memory capacity. They have to maintain information active from one source while processing the other and finally integrate both into a coherent mental representation. In a nutshell, learners’ prior knowledge and working memory capacity may well determine whether they benefit from an integrated presentation or their processing resources are over-taxed by the need to maintain information in an active state while processing other information, and by connecting both in a final step.

### The Present Study – Hypotheses

In the present study, we investigated the impact of an integrated presentation of aspects of PCK (in mathematics) and PPK. To go beyond earlier findings concerning beneficial effects of integration (i.e., information provided in an integrated way; Harr et al., 2014), we developed a further integration condition in which prompts are given in order to foster autonomous integration by the learners themselves, and compared this additional condition with the effects of “no integration” and “provided integration.” We conducted this further study to find an additional means of increasing knowledge application that (a) is more feasible in real-world settings because courses would not have to be structured in an new, integrated way, (b) may reduce cognitive load while learning, and (c) might cause less opposition from the different involved parties, because courses would not need to be merged.

We focused on two instruction-relevant components of PPK: (1) “Teaching methods” (Voss et al., 2011, p. 953; see also Borko and Putnam, 1996, p. 675: “instructional strategies for conducting lessons and creating learning environments”) and (2) “knowledge about learning processes and individual characteristics” (Voss et al., 2011, p. 953; see also Borko and Putnam, 1996, p. 675: “knowledge and beliefs about learners, how they learn and how that learning can be fostered by teaching”). For PCK, we focused on two corresponding components: (1) “Knowledge of content and teaching” (Ball et al., 2008, p. 401; see also Borko and Putnam, 1996, p. 677: “knowledge of strategies and representations for teaching particular topics”) and (2) “knowledge of content and students” (Ball et al., 2008, p. 401; see also Borko and Putnam, 1996, p. 676: “knowledge of students’ understandings [… and of] how students learn in a particular content domain”). As exemplary contents to illustrate such aspects of general PPK and PCK, we used knowledge on handling multiple external representations (MER) in classroom instruction. We provided general educational principles (e.g., general principles on how to use different representations) and domain-specific educational examples (e.g., examples on how representations are used in mathematics). The topic of flexibly using different representations plays an important role in mathematical learning and problem solving in particular (Heinze et al., 2009). In mathematics, different representations such as formulas, graphics, tables, and texts are frequently used to illustrate and deepen a topic (e.g., a fraction as a decimal number and as a piece on a pie chart). Hence, the same or overlapping information is displayed in separate representations. Normative standards for education, for example, in Germany and the USA, highlight the importance of multiple representations (e.g., National Council of Teachers of Mathematics, 2000; Ministerium für Kultus, Jugend und Sport des Landes Baden-Württemberg, 2004; National Board for Professional Teaching Standards, 2013). These standards require that teachers understand the interplay among numerical, verbal, symbolic, and graphic representations and master their transformations to facilitate students’ learning. Likewise, students should learn that a single problem may have several representations, and they should be enabled to interconnect and flexibly use them.

Beside the facilitating effects of MER (e.g., Ainsworth, 1999; Eitel et al., 2013), there is ample evidence that they may also induce high processing demands and, at worst, impair learning (Schnotz and Bannert, 2003; Ainsworth, 2006; Seufert and Brünken, 2006; Berthold and Renkl, 2009). Transition and integration processes between different representations are especially difficult (Duval, 2006). Without explicit guidance, learners often use only one representation or, at most, a few familiar ones; it is thus crucial that learners be supported (e.g., Schwonke et al., 2009).

In a former experiment (Harr et al., 2014), we had student teachers work either on an integrated computer-based learning condition or on a separate condition, addressing PPK and mathematical PCK about MER in classroom instruction. As predicted, we observed that those student teachers who had studied the provided integration condition clearly outperformed learners who had received a separate presentation in several respects.

Although the provided integration was clearly beneficial in the earlier study, it appears difficult to implement it in real-world settings. Separate university courses are the traditional means of teaching different knowledge types (Ball, 2000). Like most traditions or habits, they will not be easily abandoned. Note, however, that we assume that the benefits of integration are not due to the integrated presentation itself, but rather to the integrated encoding of different knowledge types. Thus, the separate presentation of knowledge types should not be a hindrance as long as learners engage in mental integration. Therefore, an additional means of increasing knowledge application could be to provide student teachers with specific prompts in order to trigger the mental integration that often fails to occur spontaneously when material is presented separately (Gentner et al., 2009). This approach may not only be more feasible in real-world settings – it may also reduce cognitive load while processing the respective learning content. Due to the reduced obligation to keep different information in an active and quickly retrievable state (Engle, 2002), limited working memory resources can be freed for deeper processing.

To replicate our previous findings and to determine whether such prompts may also enhance the application of educational principles, the present study compared the two conditions from the previous study with a prompted condition. The prompted group received the knowledge types in separate learning environments, plus additional questions supporting integration.

We addressed the following nine hypotheses: In a follow-up review (subsequent to the learning modules) where one condition received integration prompts, we predicted that the prompted integration group’s mental load and mental effort while working on the review exceeds the other two groups’ measures if the prompted integration group processed the prompts conscientiously (Hypothesis 1). This hypothesis can be understood as a type of manipulation check.

We predicted that mathematics student teachers are better in applying general PPK if they learn both knowledge types with provided integration or prompted integration (Hypothesis 2). For the application of PCK, we did not expect such a positive effect of integration due to its already content-related nature.

We predicted that the provided and prompted integration conditions would prove to be superior to the separated condition in simultaneously applying both knowledge types (i.e., PCK and PPK) to specific teaching problems (Hypothesis 3).

We were interested in seeing whether the integrated presentation – being more complex and demanding than separated processing – would inflict an excessive cognitive load on student teachers with low prior knowledge or low working memory capacity. We thus tested for an aptitude-treatment effect, stating that the positive effects of integration (i.e., provided and prompted integration) concerning PPK application and simultaneous application of both knowledge types are moderated by prior knowledge (Hypothesis 4) and by working memory capacity (Hypothesis 5).

We postulated four additional hypotheses with regard to cognitive demands and the efficiency of the different conditions. First, with respect to cognitive demands, we expected the provided, interleaved contents (provided integration condition) to be cognitively more demanding (while working on the learning environment) than the other two conditions so that this condition would show higher mental load when studying the learning contents (Hypothesis 6). Second, we were interested in whether the learning conditions vary in their efficiency, that is, the ability to learn a skill in minimal time (Hoffman and Schraw, 2010). We postulated three efficiency hypotheses regarding (1) PPK, (2) PCK and (3) the simultaneous application of knowledge types: (1) Although we assumed that both integration procedures facilitate learning, we expected them to be only moderately efficient with regard to PPK application due to higher learning demands caused by potentially high cognitive load (provided integration condition) and a potentially long time working with the prompts (prompted integration). Accordingly, we tested whether the integrated conditions might be less efficient than the separated group (“two-sided” Hypothesis 7a). We expected that the provided integration would be more efficient than the prompted one due to the time spent on the additional prompts (Hypothesis 7b). (2) In order to account for the possible tradeoff between PCK application and learning time, we tested whether there is a difference between conditions (“two-sided” Hypothesis 8). (3) Likewise, we tested for the simultaneous application of both knowledge types whether the integrated conditions differ from the separated condition in their efficiency (“two-sided” Hypothesis 9a). As for PPK application, we also expected that the provided integration would be more efficient than the prompted integration regarding the simultaneous application of PPK and PCK (Hypothesis 9b; for an overview of hypotheses, see **Table 1**).

**Table 1:** Overview of the hypotheses.

**Manipulation check**		
	H1:	Mental load and mental effort of the optional review Prompted integration > provided integration Prompted integration > separated condition
**Applicability of PPK and PCK**		
	H2:	Application of PPK Provided integration + prompted integration > separated condition
	H3:	Simultaneous application of both knowledge types Provided integration + prompted integration > separated condition
**Moderating effects on benefits of integration**		
	H4:	Moderation by prior knowledge
	H5:	Moderation by working memory capacity
**Cognitive demands and efficiency**		
	H6:	Mental load Provided integration condition > separated condition + prompted integration
	H7:	Efficiency regarding PPK application a: Provided integration + prompted integration ≠ separated condition b: Provided integration > prompted integration
	H8:	Efficiency regarding PCK application Provided integration ≠ prompted integration ≠ separated condition
	H9:	Efficiency regarding the simultaneous application of both knowledge types a: Provided integration + prompted integration ≠ separated condition b: Provided integration > prompted integration

## Materials and Methods

This study was conducted in accordance with the APA ethical standards (American Psychological Association, 2010) and the German Psychological Society (DGPs) ethical guidelines (Berufsverband deutscher Psychologinnen und Psychologen, 2005). The German Psychological Society’s ethical commission states that approval by an Institutional Research Board only has to be obtained if the funding is subject to the ethical approval by an Institutional Review Board. This research was reviewed and approved by the Ministry of Science, Research, and Arts of Baden-Württemberg, Germany [grant number 7532.3/130], which did not require additional Institutional Review Board approval. The Ministry of Science, Research, and Arts of Baden-Württemberg, Germany approved the procedures of this research. The participants volunteered to participate and received a compensation of 15 Euros per person. All participants were aware of taking part in a research project. Before beginning the experiment, a standardized explanation about ethical guidelines was read out loud and participants provided verbal informed consent. Participants who declined to provide the verbal informed consent were offered to withdraw from the experiment and still receive the financial reward. All participants provided written informed consent for their collected data to be used anonymously for publications. All data was anonymously collected and analyzed.

### Participants and Design

Sixty-nine mathematics student teachers (33 females, *M*_age_ = 21.84, SD = 2.28) from a German university participated in this study. Each student teacher received 15 Euros for participation and took part in a prize draw to win a mini iPad. The students were randomly assigned to one out of three conditions which differed in terms of the integration of learning contents. The first condition treated both knowledge types (i.e., PCK and general PPK) successively in a separate way (separated condition, *n* = 23). The second and third conditions, however, provided the knowledge types in integrated, connected ways. In the second condition, the knowledge types were presented together and interleaved (provided integration, *n* = 23). In the third condition, student teachers received prompts on how contents should be integrated (prompted integration, *n* = 23). The central dependent variables referred to the application of general PPK, PCK, and the combined use of knowledge types. One participant in the prompted-integration condition was excluded from further analyses for having behaved inappropriately during the experiment (e.g., providing nonsense answers).

### Materials and Procedure

#### Working Memory Task

In order to assess each student teacher’s working memory spans, we applied a working memory task, designed in accordance with Unsworth et al. (2005). The student teachers first read a short sentence and then classified it as being either sensical or nonsensical. Second, they were asked to remember a set of unrelated letters presented behind each sentence (e.g., “The infant suffered from an ear infection and therefore had to stay in the lettuce for 3 weeks. X”). We set a threshold of 85% accuracy in sentence-classification for working memory scores to be valid. Due to this threshold, the scores of three participants were excluded in further analyses that incorporated working memory scores. The threshold ensured that participants did not focus on remembering the letters while ignoring the first task (i.e., reading and classifying the sentences). Sensical and nonsensical sentences were balanced out, while sentence length varied between 10 and 15 words. In order to obtain nonsensical sentences, we replaced one word in an otherwise sensical sentence with an incongruous word (e.g., “lettuce” instead of “bed”). The sentences were arranged in different set sizes: one set encompassing two to five cascaded sentences. Once participants had finished one set, they were asked to recall the previously presented letters in their original order. The students completed three laps with varying sentences for each set size. They were given one point for each correctly retrieved letter if it was also in the correct position.

#### Pretest

The pretest assessed prior PCK and general PPK on MER. The measure included six open-ended questions, three out of which were tapping on each knowledge type (e.g., PPK aspects: “Please name three functions that can be fulfilled by using multiple representations”; PCK aspects: “Please name four arbitrary representations (also graphic) for fractions”). In a blind coding, the answers were scored in accordance with a previously developed coding scheme assessing aspects of PCK and PPK about learning from MER. This initial coding scheme was based on expert answers which were then broken down into single statements. However, in order to obtain a better fit to student answers, the scheme was slightly modified from the initial scheme as used in an earlier study. Participants were awarded one point for each correctly mentioned aspect (e.g., one arbitrary representation named). Twenty percent of the questions were scored by a second, independent rater (not adjusted *ICC* = 0.97). Disagreements were resolved through discussion.

As the maximum score varied between items, we performed a *z*-standardization on PPK and PCK scores in order to equalize weights before aggregating them into the pretest scale. The PPK score showed very low reliability (*Cronbach’s* α = 0.17), which was probably caused by the participants’ very low PPK knowledge (i.e., on average just 1.47 out of 12 points were gained as raw score). As the PCK score proved to be sufficiently reliable (*Cronbach’s* α = 0.81), we used this score as the pretest measure in the following analyses.

#### Learning Environments

Student teachers were randomly assigned to one out of three conditions: separated, provided integration, or prompted integration. Each condition provided information on MER from a general pedagogical and psychological (i.e., aspects of PPK) and a mathematics-education stance (i.e., aspects of PCK in the domain of fractions). Thus, the essential difference between conditions was that PPK and PCK on MER were treated either in an integrated way (i.e., provided or prompted) or apart from each other.

For the pedagogical and psychological learning contents (i.e., PPK learning environment), we focused on more general functions and aspects of MER that should be kept in mind when working with MER (e.g., text, tables, graphs). We addressed psychological functions of MER (e.g., the constraining function stating that one representation can constrain the interpretation of another one). We addressed means of adequate support for students on the surface-feature level (e.g., color-coding) and on a deep-structure level (i.e., explicitly explaining the relations between corresponding structures). We further addressed the cognitive demands of constructing a coherent mental model from MER. Information on the pedagogical and psychological stance was based on literature by Ainsworth (1999, 2006), Seufert and Brünken (2006); Bodemer (2008), Rau et al. (2009) and Schwonke et al. (2009).

For the mathematics education learning contents (i.e., PCK learning environment), we focused on several didactical aspects that should be considered when working with MER. We addressed pitfalls of translation between different representations of fractions (e.g., the difficulty students have with labeling hatched areas in a model with the correct fraction). We addressed different aspects of fraction numbers (e.g., fraction numbers as fraction or as ratio). As a strategy for teaching particular topics, we further addressed a mathematics education principle quite popular in Germany, namely the enactive-iconic-symbolic principle (i.e., EIS principle incorporating enactive, iconic and symbolic representations). The EIS principle is part of almost all German mathematics education textbooks. Information on the mathematics education learning contents paralleled the respective German mathematics education literature applied later in the participants’ teacher education programs (Wittmann, 1981; Hasemann, 1986; Hefendehl-Hebeker, 1996; Zech, 1996; Padberg, 2009; Eichelmann et al., 2012).

In a nutshell, the main difference between both stances (i.e., pedagogical and psychological learning contents and mathematics education learning) was the perspective on MER. The mathematics education stance provided a mathematics-specific view of MER fractions (i.e., pitfalls of fraction representations, different aspects of fraction numbers, EIS principle). The pedagogical and psychological stance focused on a more general view of MER (i.e., psychological functions of MER, means of supporting students on different levels, cognitive demands of constructing coherent mental models) by using exemplary illustrations of everyday life (e.g., genesis of Fata Morganas). Due to the rationale of combining general knowledge with mathematics-specific examples, an overlap of both stances was created in the provided integration condition by modifying examples of the pedagogical and psychological stance in order to refer to fractions.

**FIGURE 1:**
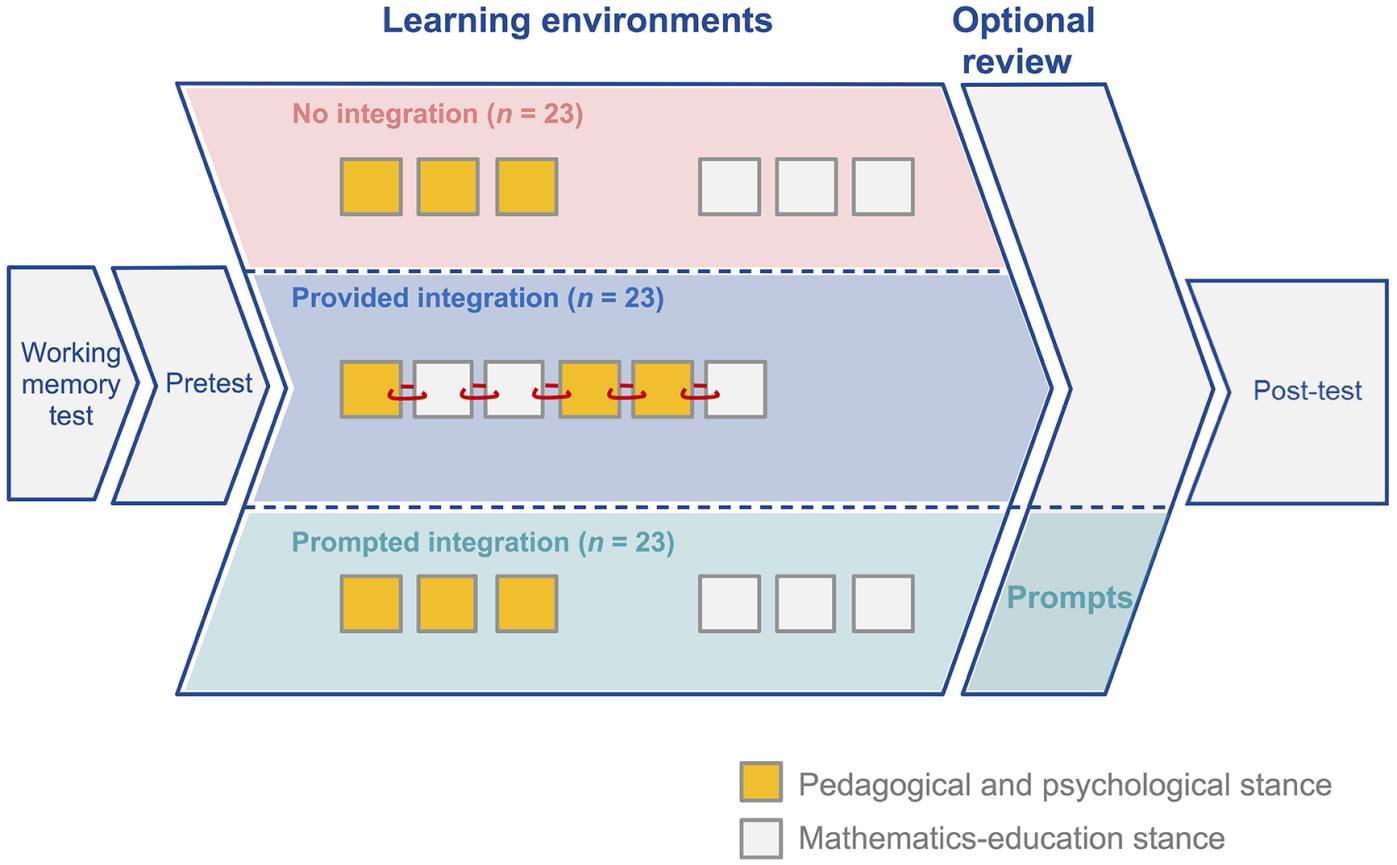
**Overview of the procedures of the three experimental conditions**.

**FIGURE 2:**
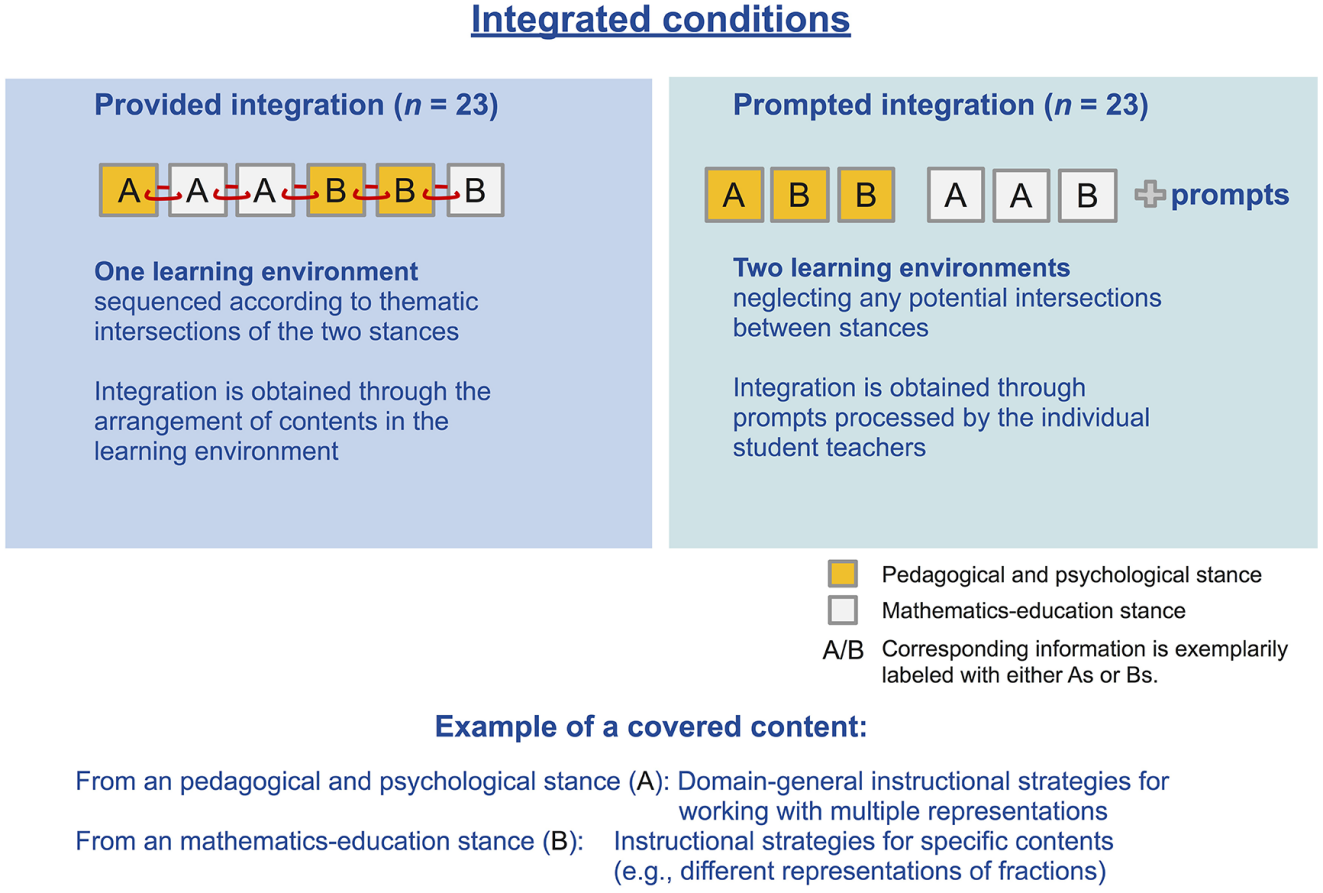
**Illustration of how integration of contents was achieved in the different integration conditions**.

The learning conditions consisted of one (longer) or two (shorter) learning environments. Both the separated and prompted integration condition consisted of two learning environments (i.e., the pedagogical and psychological stance and mathematics-education stance). Due to a lack of sequencing effects in the prior study (Harr et al., 2014), the order of environments was fixed. Student teachers of the separated and prompted integration condition worked first on the pedagogical and psychological and then on the mathematics educational environment. The provided integration condition comprised only one learning environment which combined the contents of the two learning environments. In order to achieve smooth integration, the slides were arranged in a thematically coherent sequence and examples were altered (for an overview of conditions, see **Figure 1**). In order to smoothly integrate different topics (i.e., the slides containing either PPK or PCK), we added a few connecting phrases. These phrases were not included in the separated and prompted condition. However, the number of words and basic information were kept constant by deleting some filler words in the provided integration condition – balancing for the additional transition sentences. Note that the additional transition sentences yielded no supplementary information on multiple representations (e.g., “Before the functions will be explained in detail, a brief introduction to the EIS principle will be provided”; for an overview of the means of integration, see **Figure 2**). The student teachers could not go back and forth in the learning environments to ensure a fixed order of information processing for all participants. They were asked to proceed at their own pace.

#### Worksheet Presenting the Prompts

In the prompted integration condition, a worksheet briefly informed the student teachers about the importance of not just understanding the learned contents separately (i.e., how they were presented in this condition), but of drawing connections between topics in order to achieve a deep understanding. Following this brief initial explanation, they were asked to process several prompts focusing on the connections between different topics in the learning environments (e.g., “Please reflect on how the representations of the EIS principle/of fractions are connected to the MER functions that were addressed in the pedagogical and psychological learning environment. Why can the constraining function be fulfilled through the EIS principle?”).

#### Optional Review

After the learning phase, student teachers were given the opportunity to review the learning contents on six optional wrap-up slides. The purpose of the optional review was to ensure a sound basis for integration processes in the prompted condition. However, in order to equalize this component across groups, we included it in all three learning conditions. Three slides dealt with MER functions, cognitive overload, and support, and three other slides dealt with issues concerning the EIS principle, implications, and implementation. The instructions differed slightly, depending on the condition. Students in the separated and the provided integration conditions were instructed to click on the respective slide if they wanted to review the learning contents. Eleven student teachers of the separated condition and 16 student teachers of the provided integration condition used the slides. Students in the prompted integration condition were asked to take a look at the worksheet presenting the prompts. They were also told they could use the on-screen slides for help. All student teachers of the prompted integration condition used the slides in order to review the information given in the learning environment.

#### Post-Test

The post-test consisted of 10 rapid assessments and 12 open-ended questions. We designed the rapid assessments in accordance with Kalyuga’s rapid verification method – a valid and time-saving approach for knowledge assessment (e.g., Kalyuga, 2006a,b, 2008). Here, learners were required to either verify or falsify presented statements within a short period of time. The verification statements referred to descriptions of teaching situations given beforehand. Each description was displayed for a limited time and immediately followed by verification statements (e.g., a teaching situation was described in which a teacher and her students sort paperclips by color, then discuss different graphic representations, and finally talk about ways to represent the distributions in tables; one rapid verification item tested, for example, whether or not the student teachers recognized the central purpose from the pedagogical and psychological stance: “The deep understanding function (3) is fulfilled through different representations”). We constructed verification items for mathematical PCK and general PPK.

The student teachers completed this task individually. Different elements should be combined in accordance with the principles taught in the preceding learning environment(s).

**FIGURE 3:**
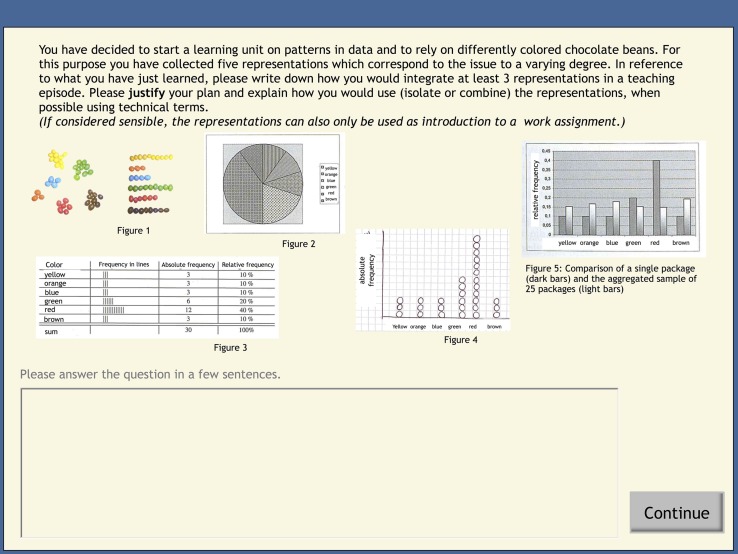
**Screenshot of a mathematics-classroom scenario from the post-test (translated from German).** The student teachers completed this task individually. Different elements should be combined in accordance with the principles taught in the preceding learning environment(s).

**FIGURE 4:**
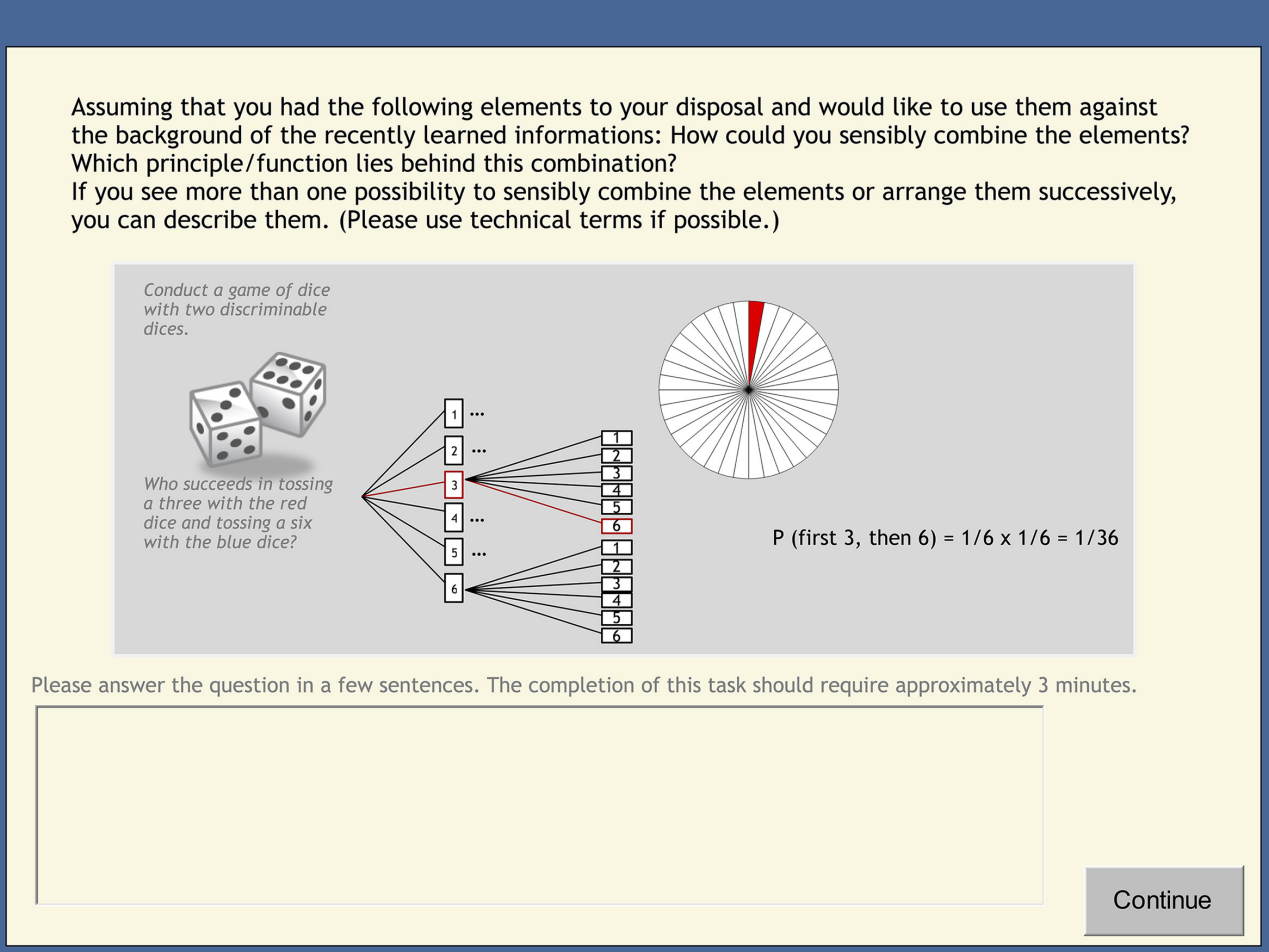
**Screenshot of a mathematics-classroom scenario from the post-test (translated from German).** Student teachers were asked to combine the elements in accordance with the taught principles of the learning environment(s).

The open-ended questions consisted of two parts. Five open-ended questions asked for declarative knowledge on the topics, such as listing different possibilities of mathematical representations or naming functions of multiple representations. Seven open-ended questions included mathematics-classroom scenarios (e.g., in one scenario, students assumed the role of a teacher who was planning to start a learning unit on patterns in data. The ‘teacher’ had already decided to use colored chocolate beans for illustration and altogether had five tables and illustrations from which she was asked to use at least three when sketching her first teaching episode in the unit. Participants were asked to justify their course of action; for further details, please see **Figure 3**; for an additional post-test item example, please see **Figure 4**.). These scenarios addressed the subdomain “data and chance” that differs from the learning subdomain (i.e., fractions) and is fairly novel in German schools (e.g., Eichler and Vogel, 2009). In order to answer the questions, students could use PCK or general PPK, or both knowledge types. In order to compute the post-test score, we used a previously developed blinded coding system to assess general PPK and PCK. For the seven teaching scenarios, the coding scheme was based on expert answers comprising aspects previously addressed in the learning environments of PPK (e.g., MER functions or helping strategies) and PCK (e.g., typical pitfalls with different representations or the EIS principle). Expert answers were subsequently broken down into single statements which could be mentioned separately addressing different aspects of knowledge (e.g., one specific function of MER). However, in order to obtain a better fit on student answers, we slightly modified an initial scheme from an earlier study. Participants were awarded one point for every sensibly-mentioned aspect. Two scores were created. More general pedagogical and psychological statements on MER (e.g., MER functions) were coded as PPK [e.g., “The students could draw relations between the elements and, through this, foster understanding. Additionally, one representation can constrain or complement another one (two MER functions)”]. Mathematical PCK statements (e.g., typical pitfalls or the EIS principle as a strategy for teaching a particular content) were coded as PCK [e.g., “Students learn first through concrete acting (enactive form), which is then transformed into the iconic form by using diagrams and later transformed into the symbolic form by setting up probabilities”]. As already pointed out, one point was awarded for each correctly mentioned aspect. However, students received no extra points for repeatedly mentioning the same aspect with a different wording in response to the same question. Twenty percent of the post-test questions were scored by a second rater (not adjusted *ICC* = 0.86). Disagreements were resolved through discussion.

The number of aspects students could mention when answering the open-ended questions varied between items. The maximum scores ranged from three to six points. In order to cope with this variation in maximum scores, we *z*-standardized all items in order to equalize weights before aggregating them into the scales.

The dependent variables on application of general PPK (*Cronbach’s* α = 0.72) and PCK (*Cronbach’s* α = 0.68) were obtained by taking the PPK and PCK scores of the seven teaching scenarios. Due to the low reliability of rapid assessment items (i.e., PCK, *Cronbach’s* α = 0.22 and PPK, *Cronbach’s* α = 0.52), we abstained from calculating a combined score. Simultaneous use of knowledge types, the third dependent variable, was based on a computation in which one point was awarded when student teachers solved a teaching scenario by using the respective components of PPK (e.g., MER functions) and PCK (e.g., typical pitfalls or the EIS principle as strategy for teaching a particular content) together.

In order to gain further insight into learning outcome relative to learning time invested, we calculated a respective efficiency measure (Hoffman and Schraw, 2010). We defined efficiency as learning outcome (*z*-standardized measure of PPK application) divided by the learning time (*z*-standardized measure). In order to avoid negative efficiency scores (that made little sense) due to negative *z*-values, we added a constant of +3 to the data when determining the efficiency scores.

#### Mental Load and Mental Effort

Mental load and mental effort are considered to be two aspects of cognitive load (Paas and van Merriënboer, 1994). Mental load is defined as the cognitive load stemming from the interaction of task- and learner-related variables, however, mainly associated with the task (Paas et al., 2003; van Gog and Paas, 2008). Mental effort indicates the cognitive load actually invested while solving a task (Paas and van Merriënboer, 1994; Paas et al., 2003). We measured the subjective task difficulty (i.e., mental load) four times in the learning environment. In relation to the optional review, we additionally measured invested effort (i.e., mental effort). The scale ranged from 1 (mental load: *not at all difficult*; mental effort: *no effort at all*) to 10 (mental load: *very difficult*; mental effort: *very high effort*). The four items in the learning environment were combined to form an overall score on mental load (*Cronbach’s α* = 0.77).

#### Procedure

All student teachers first received a brief introduction on how to handle the computer program and then proceeded to the on-screen working memory task. Afterward, a pretest assessed general PPK and PCK. The student teachers were then informed about the topics of the upcoming computer-based learning contents and about the final post-test questions. They were told they could proceed at their own pace so that they could work toward really understanding the contents. The participants were also informed that they could monitor their progress in the learning environment by inspecting a bar in the upper left corner of each slide. The student teachers then began working on the learning conditions and ultimately completed the post-test (for an overview of the procedure, see **Figure 1**).

### Results

There is one table for each set of hypotheses showing the means and standard deviations of the central variables for all three conditions. An alpha level of 0.05 was used for all statistical analyses. As an effect size measure, we used partial $\eta_{p}^{2}$. According to Cohen (1988), we regarded values <0.06 as small effects, values in the range between 0.06 and 0.13 as medium and values >0.13 as large effects.

#### Pre-Analysis and Manipulation Check

The experimental groups did not differ in prior knowledge, working memory capacity, and demographic variables such as age, number of pedagogy courses at university, number of semesters, mother tongue, and gender (all *p*s > 0.10). In order to ensure that student teachers in the prompted integration condition had processed the additional prompts during the review slides, we carried out a manipulation check. In Hypothesis 1, we assumed that the prompted integration group’s mental load and mental effort while working on the optional review would exceed the other two groups’ measures if the prompted integration group had processed the prompts conscientiously (**Table 2**). Planned contrasts revealed that the mental load was rated significantly lower in the separated condition, *p* < 0.001, 95% CL [4.41, 2.33] and in the provided integration condition, *p* < 0.001, 95% CL [3.33, 1.24] as compared to the prompted integration condition. The same pattern was established in terms of mental effort. Planned contrasts demonstrated that mental effort was rated significantly lower in the separated condition, *p* < 0.001, 95% CL [4.26, 1.94] and in the provided integration condition, *p* < 0.001, 95% CL [3.74, 1.42] than in the prompted integration condition. We can hence assume that the prompted integration group processed the prompts.

**Table 2:** Mean (and SD) of mental load and mental effort while working on the optional review (and on the prompts for the promoted integration condition respectively) in the three experimental groups.

	Separated condition	Provided integration condition	Prompted integration condition
Mental load (optional review)	3.17 (1.50)	4.26 (1.94)	6.55 (1.79)
Mental effort (optional review)	4.22 (1.98)	4.74 (2.36)	7.32 (1.36)

*Scales ranged from 1 (mental load: *not at all difficult*; mental effort: *none effort at all*) to 10 (mental load: *very difficult*; mental effort: very high effort)*.

### Central Hypotheses

We predicted in Hypothesis 2 that mathematics student teachers are better at applying general PPK if they learned PCK and general PPK in a provided or prompted integration condition rather than in the separated condition. Planned contrasts revealed that the integrated conditions enhanced the applicability of PPK, *t*(65) = 2.71, *p* = 0.005 (one-tailed), $\eta_{p}^{2}$ = 0.10 (**Table 3**). We thus confirmed Hypothesis 2; student teachers in the integrated conditions applied more PPK. The provided and prompted integration conditions did not differ significantly, *t*(65) = 0.53, *p* = 0.601 (two-tailed), $\eta_{p}^{2}$ = 0.00. We also conducted a further analysis (ANOVA) in order to see whether integration exerts effects on the applicability of PCK (**Table 3**), but detected no significant effect regarding the application of PCK, *F*(2,65) = 0.96, *p* = 0.387, $\eta_{p}^{2}$ = 0.03.

**Table 3:** Mean (and SD) of z-standardized PPK and PCK application and the simultaneous application of both knowledge types in the three experimental groups.

		Separated condition	Provided integration condition	Prompted integration condition
Post-test	General pedagogical and psychological knowledge application	-0.44 (0.79)	0.15 (0.93)	0.30 (1.14)
	Pedagogical content knowledge application	0.16 (1.21)	-0.23 (1.01)	0.08 (0.71)
	Combined use of knowledge	-0.37 (0.92)	-0.01 (0.97)	0.40 (1.00)

Hypothesis 3 addressed the capability to adopt multiple perspectives on teaching tasks. We predicted that the provided and prompted integration conditions would prove to be superior to the separated condition in simultaneously applying both knowledge types (i.e., PCK and PPK) to specific teaching problems (**Table 3**). Planned contrasts revealed that the integrated conditions showed a greater simultaneous application of both knowledge types than the separated condition, *t*(65) = 2.27, *p* = 0.014 (one-tailed), $\eta_{p}^{2}$ = 0.07, and that provided and prompted integration did not differ significantly, *t*(65) = 1.41, *p* = 0.162 (two-tailed), $\eta_{p}^{2}$ = 0.03. Hypothesis 3 was therefore confirmed as well. Providing student teachers with an integrated view on PCK and PPK increased the simultaneous application of knowledge types as compared to the separated condition where student teachers rather focused on one at a time.

Hypotheses 4 and 5 both predicted that integration benefits would be moderated by specific person-related variables (**Table 4**). We tested for an aptitude-treatment effect of prior knowledge (Hypothesis 4) and working memory capacity (Hypothesis 5). However, we identified no moderating effects of prior knowledge on either the application of PPK [interaction term: *F*(1,64) = 0.46, *p* = 0.499, $\eta_{p}^{2}$ = 0.01] or on the simultaneous application of knowledge types [interaction term: *F*(1,64) = 0.00, *p* = 0.955, $\eta_{p}^{2}$ = 0.00]. The same applies to working memory: we detected no moderating effects on the application of PPK [interaction term: *F*(1,61) = 0.76, *p* = 0.386, $\eta_{p}^{2}$ = 0.01] or on the application of both knowledge types [interaction term: *F*(1,61) = 1.55, *p* = 0.218, $\eta_{p}^{2}$ = 0.03]. Thus, Hypotheses 4 and 5 were both rejected. Student teachers benefited from the integrated conditions, regardless of prior knowledge and working memory capacity.

**Table 4:** Mean (and SD) of the z-standardized pretest and working memory scores in the three experimental groups.

		Separated condition	Provided integration condition	Prompted integration condition
Pretest	General pedagogical and psychological knowledge	-0.08 (0.80)	0.07 (1.12)	0.01 (1.09)
	Pedagogical content knowledge	0.05 (1.12)	-0.21 (0.69)	0.17 (1.13)
Working memory task		37.95 (3.34)	39.43 (2.06)	38.91 (2.89)

*The attainable maximum score of the working memory task was 42 points*.

Hypothesis 6 addressed the cognitive challenge of interleaved learning contents in the provided integration condition. We predicted that the provided integration condition would induce higher mental load than the prompted and separated conditions when studying the learning environment (**Table 5**). Planned contrasts revealed that a provided integration of learning contents increased mental load when working on the learning environment as compared to the separated or prompted conditions, *t*(65) = 1.76, *p* = 0.042 (one-tailed), $\eta_{p}^{2}$ = 0.05. The separated and prompted conditions did not differ significantly in this respect, *t*(65) = 0.38, *p* = 0.703 (two-tailed), $\eta_{p}^{2}$ = 0.00. Thus, Hypothesis 6 was confirmed. The provided integration condition induced higher mental load when processing new learning material than did the separated or prompted integration conditions.

**Table 5:** Mean (and SD) of mental load while working on the learning environment in the three experimental groups.

	Separated condition	Provided integration condition	Prompted integration condition
Mental load (learning environment)	4.62 (1.50)	5.23 (1.71)	4.44 (1.39)

*The scale ranged from 1 (mental load: *not at all difficult*) to 10 (mental load: very difficult)*.

Hypotheses 7, 8, and 9 addressed learning efficiencies (outcome/learning time; **Table 6**). In a first step, we compared the learning times of the separated, provided, and prompted integration conditions (**Table 6**). We found that the separated condition demanded less time than the integrated ones [*t*(56.25) = 6.76, *p* < 0.001 (two-tailed), $\eta_{p}^{2}$ = 0.45] and that the provided integration condition demanded less time than the prompted condition, *t*(34.26) = 5.96, *p* < 0.001 (two-tailed), $\eta_{p}^{2}$ = 0.51. Hypothesis 7 addressed the learning efficiency of PPK application (**Table 6**). We tested whether the integrated conditions might be less efficient than the separated group (Hypothesis 7a). Planned contrasts revealed that the two integrated conditions were just about as efficient as the separated group, *t*(65) = 0.72, *p* = 0.474 (two-tailed), $\eta_{p}^{2}$ = 0.01. We expected that the provided integration is more efficient than the prompted integration, due to the time spent on additional prompts (Hypothesis 7b). In line with our prediction, we found that regarding PPK application, the provided integration condition was more efficient than the prompted integration condition, *t*(65) = 3.34, *p* = 0.001 (one-tailed), $\eta_{p}^{2}$ = 0.15, thus confirming Hypothesis 7b.

**Table 6:** Mean (and SD) of learning time and efficiencies in the three experimental groups.

	Separated condition	Provided integration condition	Prompted integration condition
Learning time in minutes	24.99 (5.73)	29.01 (7.35)	46.96 (12.16)
Efficiency PPK	1.12 (0.37)	1.24 (0.44)	0.86 (0.34)
Efficiency PCK	1.36 (0.49)	1.07 (0.39)	0.80 (0.26)
Efficiency combined use of knowledge	1.14 (0.37)	1.17 (0.44)	0.90 (0.37)

*Learning time ranged from 12 to 68 min*.

Hypothesis 8 addressed the learning efficiency of PCK application (**Table 6**). In order to account for possible tradeoffs between PCK application and learning time, we tested for any differences between the conditions. Planned contrasts showed that the two integrated conditions were less efficient than the separated group [*t*(32.51) = 3.69, *p* = 0.001 (two-tailed), $\eta_{p}^{2}$ = 0.30] and that the provided integration condition was more efficient than the prompted integration condition, *t*(38.46) = 2.78, *p* = 0.008 (two-tailed), $\eta_{p}^{2}$ = 0.17. Thus, Hypothesis 8 was also confirmed.

Hypothesis 9 addressed the learning efficiency of the simultaneous application of both knowledge types (**Table 6**). We tested whether the integrated conditions differed in their efficiency from the separated condition (Hypothesis 9a). Planned contrasts revealed that the two integrated conditions were just as efficient as the separated group, *t*(65) = 1.01, *p* = 0.319 (two-tailed), $\eta_{p}^{2}$ = 0.02. As for PPK application, we also expected for the simultaneous application of PPK and PCK that the provided integration is more efficient than the prompted integration (Hypothesis 9b). In line with our prediction, we noted that the provided integration condition was more efficient in applying both knowledge types than the prompted integration condition, *t*(65) = 2.36, *p* = 0.011 (one-tailed), $\eta_{p}^{2}$ = 0.08.

## Discussion

We investigated the integration of knowledge types that are usually separated in teacher training (Ball, 2000) and proposed a possible route to support the applicability of PPK aspects relevant in teaching subjects (Voss et al., 2011). We provided student teachers with learning conditions treating knowledge types in either a separate or an integrated way (provided or prompted) in order to foster combined encoding (Colhoun et al., 2008; Renkl, 2014). Drawing on our previous research, we derived several hypotheses concerning possible means of fostering the application of knowledge and moderating variables as well as efficiency measures. The results of testing these hypotheses are summarized below.

From our overall analyses, we conclude that our study has successfully replicated the positive effects of provided integration we had observed in a previous study (Harr et al., 2014). More specifically, the applicability of PPK aspects was enhanced by the integrated presentation of knowledge types. Moreover, we demonstrated in this study that prompted integration was also effective. The student teachers in the two integrated conditions earned higher application scores on PPK. When considering only the outcome values, we observed no losses in the investigated PCK aspects. However, when taking efficiency into account, we did identify detrimental effects associated with PCK application, which will be discussed later. Still, one major implication of this study is that the application of the addressed components of PPK can be fostered through integration.

Integration also fostered the simultaneous application of aspects of both knowledge types, which is consistent with earlier findings (Harr et al., 2014). Accordingly, receiving an integrated view on knowledge types facilitates the flexible use of both perspectives. However, as in the case of PCK application, this positive effect was achieved at the expense of increased learning time. Aspects of efficiency will be discussed later in more detail.

In contrast to our predictions but in line with earlier results, we found no moderating effects of prior knowledge or working memory capacity. Neither low prior knowledge nor low working memory capacity undermined the beneficial effects of the integrated conditions. The effects of the different conditions were thus similar to different degrees of prior knowledge or working memory capacity. The encouraging news is that integration is feasible without overwhelming students with low prior knowledge or low working memory capacity.

Finally, in line with our predictions, the provided integration condition inflicted a significantly higher mental load in the learning environment than the other conditions. When comparing the conditions regarding the learning efficiency of PPK application, the integrated conditions were as efficient as the separated condition. However, the provided integration exceeded the prompted condition significantly. Regarding the learning efficiency of PCK application, we observed that the two integrated conditions were less efficient than the separated group and that the prompted integration condition was even less efficient than the provided integration. Concerning the learning efficiency of the simultaneous application of both knowledge types, as for PPK application, the two integrated conditions were as efficient as the separated group and the provided integration exceeded the prompted condition significantly.

Overall, the pattern of findings with respect to learning outcomes and efficiency can be interpreted as follows. Provided integration leads to better outcomes than separate presentation with respect to PPK and the simultaneous use of PPK and PCK. These positive effects are achieved through a moderate increase (of roughly 16%) in required learning time (see **Table 6**). There are no negative side effects with respect to PCK outcomes. However, when taking the longer learning time into account, one could argue that the students need a bit more time (16%) to reach the same level of PCK. When comparing prompted integration to separate presentation, we attained comparable outcome advantages to the provided integration. However, prompted integration is associated with a substantial drawback in terms of efficiency (roughly an 88% increase in learning time; see **Table 6**).

Against the background of the pattern of findings, prompted integration does not seem to be a sensible option at first glance, given its limited efficiency. At a second glance, we see a potential dilemma between the efficiency and feasibility of the different integration modes – provided integration is more efficient, yet difficult to implement in real-world settings. The need would arise to restructure curricula offering courses that treat subject-related matter and general educational principles together. Hence, the provided integration approach is more efficient but might be difficult to implement. By contrast, the prompted integration takes more learning time. However, its implementation in real-world settings is readily feasible, because curricula need not undergo major modification, while integration would be prompted through special homework assignments or tutorials. Another advantage of prompted integration is that (due to its separate introduction of subjects) it is cognitively less demanding if students encounter new learning contents. Thus, prompted rather than provided integration has the advantages of greater feasibility and lower cognitive demands (i.e., cognitive load) but also the disadvantage of increased learning time. To sum this up, prompted integration shows several benefits: (a) Other than for the provided integration, university courses would not need to be restructured and only some additional tutorials (or even only respective homework) would have to be added to the curricula of teacher education. (b) Prompted integration is cognitively less demanding for student teachers and should thus be carried out without mayor difficulties. (c) In contrast to the provided integration, implementation would be easier because prompted integration can be assumed to receive less opposition from involved parties due to the fact that courses need not be merged and instructors can retain their proper courses.

Our findings demonstrate sensible approaches toward improving the integration of different knowledge types. Our results can thus be regarded as initial evidence that the usually strict separation of courses in teacher-education programs might be less than ideal, at least when PCK and PPK aspects can be sensibly interrelated. However, it should be noted that these findings might only be directly applicable for the PPK and PCK aspects considered in this study. For other important aspects of PPK such as knowledge about classroom management, the present rationale of integrated encoding with PCK does not appear to be applicable. Overall, knowledge about classroom management might be easier to apply in different subject areas than knowledge on teaching with MER, because there is less need to tailor the general principles (of classroom management) to the contents.

In line with other findings (e.g., Bandura, 1986; Colhoun et al., 2008; Holyoak, 2012; Renkl, 2014), our results highlight the need to bring different parts of knowledge together. It is quite surprising that university courses are usually taught as separate units addressing pedagogy and content-related information, leaving student teachers with the task of integration (Ball, 2000). In line with other research (e.g., Renkl et al., 1996; Ball, 2000; Grossman et al., 2009; Goldstone and Day, 2012; Seidel et al., 2013), we see a great need to foster the applicability of knowledge in the educational context. However, it seems that even today, the need for applicable knowledge is failing to encourage a change in practice. Students are often still left to integrate knowledge on their own, which, in turn, hampers their acquisition of applicable PPK.

In summary, this study yields insights on three major issues beyond our previous study (Harr et al., 2014). First, we successfully replicated our earlier study’s results concerning the applicability of knowledge types and possible detriments stemming from integration demands. Second, in addition to the previous study’s experimental conditions, we introduced a supplemental condition. This prompted integration condition proved to be effective as well. Third, we disclosed only moderate efficiency of the integrated conditions. Although the provided integration turned out to be a more efficient integration method regarding PPK application and the simultaneous application of both knowledge types, the integrated conditions did not exceed the learning efficiency of the separated conditions. Thus, the benefits of integration were narrowed when taking learning time into account. With PCK application, we even observed detrimental effects of integration when also considering learning time. Thus, unfortunately, we are unable to claim that integration has no drawbacks in terms of PCK application when learning time is taken into account.

### Limitations and Directions for Further Research

Despite these promising insights on the applicability of PPK aspects, some restrictions and open questions should be addressed. The present findings were obtained from a short-term experimental design, and the effects are mostly of medium size. Long-term interventions may well yield more profound effects. Before studies testing extended interventions are available, implications for long-term processes in teacher education must be drawn with caution. The present study should be regarded as an initial step toward further investigations of integration in real university courses. However, our approach’s limitation also bears a potential strength: due to the randomization process and standardized instructions, confounding variables can be controlled and effects attributed to experimental variation.

A further limitation concerns the use of only brief templates of teaching situations in order to assess knowledge applicability. Through these templates, we tested the student teachers’ ability to apply knowledge across different situations. However, the study did not test application in real lesson planning or classroom teaching.

Another issue is that the EIS Principle we introduced as one of the contents of the PCK learning environment (i.e., as a strategy for teaching particular topics following the PCK components by Borko and Putnam, 1996) might also possess general aspects, that is, it might be more broadly applicable even though it is commonly found in the mathematics education curriculum. Due to these general aspects, this component might have been transferred more easily from the learning topic (i.e., fractions) to the post-test topic (i.e., data and chance) and might therefore account for missing post-test differences between conditions in PCK. However, we observed a clear distance from the maximum (*M* = 11.01, SD = 4.01, score_max_ = 32; no ceiling effect), which suggests a sufficient difficulty of PCK transfer.

Another potential restriction is the implementation of PPK aspects in the provided integration condition. Other than in the separated condition where PPK illustrations referred to everyday life examples (e.g., the genesis of Fata Morganas), examples referred to fractions, thus creating an overlap between stances. This overlap followed the rationale of combining general knowledge with mathematics-specific examples in order to attain a genuine integration of contents. However, via this integration method, the provided integration condition did not only integrate through proximity of contents but also via content-specific integration. Varying this integration method might be worthwhile to investigate the pure effect of interleaving. In this variation, integration should only be accomplished through proximity (i.e., integration of the slides for PPK and PCK) and not through content-specific integration (i.e., a change in examples from everyday life to the mathematics subject).

In summary, it would be interesting to investigate the following issues in further research: (a) implementation of a modified provided integration condition could be tested in order to gain further insight into integration processes; (b) in order to complement our present results, identical learning times could be maintained across all conditions; (c) we see an obvious need to extend our experimental approach to longitudinal field study designs situated in real teacher-education contexts. Additionally, future research should also be extended to other subject domains and disciplines.

To our knowledge, integrated approaches regarding PCK and PPK integration are yet missing. However, TPACK research has already rendered encouraging results concerning merits of integration (e.g., Koehler et al., 2007; Niess et al., 2010). In our case, the prompted integration approach seems to be particularly feasible for implementation in real teacher education, because prompts for integration of courses could be given as homework or supported by tutorials without major changes in the given curriculum. Students of various subject areas could attend the same subject-independent courses and subsequently – with the respective prompts – draw connections to their specific subjects. The results of the present study show that drawing connections between pedagogical and psychological principles and content-specific educational knowledge fosters the application of some type of PPK. Furthermore, integration yielded significant benefits for applying both pedagogical and psychological principles and educational knowledge. The additional time cost of integration could be buffered by outsourcing integration from the courses itself, either as homework assignments or in additional tutorials. In this regard, our study provides a sound basis for further *in vivo* longitudinal designs on the applicability of PPK.

## Conflict of Interest Statement

The authors declare that the research was conducted in the absence of any commercial or financial relationships that could be construed as a potential conflict of interest
